# BioWord: A sequence manipulation suite for Microsoft Word

**DOI:** 10.1186/1471-2105-13-124

**Published:** 2012-06-07

**Authors:** Laura J Anzaldi, Daniel Muñoz-Fernández, Ivan Erill

**Affiliations:** 1 Department of Biological Sciences, University of Maryland Baltimore County, 1000 Hilltop Circle, Baltimore, MD, 21250, USA; 2 Departament de Ciències de la Computació, Universitat Autònoma de Barcelona, Campus UAB s/n, Bellaterra, Barcelona, 08193, Spain

## Abstract

**Background:**

The ability to manipulate, edit and process DNA and protein sequences has rapidly become a necessary skill for practicing biologists across a wide swath of disciplines. In spite of this, most everyday sequence manipulation tools are distributed across several programs and web servers, sometimes requiring installation and typically involving frequent switching between applications. To address this problem, here we have developed BioWord, a macro-enabled self-installing template for Microsoft Word documents that integrates an extensive suite of DNA and protein sequence manipulation tools.

**Results:**

BioWord is distributed as a single macro-enabled template that self-installs with a single click. After installation, BioWord will open as a tab in the Office ribbon. Biologists can then easily manipulate DNA and protein sequences using a familiar interface and minimize the need to switch between applications. Beyond simple sequence manipulation, BioWord integrates functionality ranging from dyad search and consensus logos to motif discovery and pair-wise alignment. Written in Visual Basic for Applications (VBA) as an open source, object-oriented project, BioWord allows users with varying programming experience to expand and customize the program to better meet their own needs.

**Conclusions:**

BioWord integrates a powerful set of tools for biological sequence manipulation within a handy, user-friendly tab in a widely used word processing software package. The use of a simple scripting language and an object-oriented scheme facilitates customization by users and provides a very accessible educational platform for introducing students to basic bioinformatics algorithms.

## Background

In a relatively short time, editing and processing of DNA and protein sequences have left the realm of molecular biology to become a routine practice for biologists working in myriad different fields. At the same time, the number of tools and servers for performing analyses on biological sequences and related data has exploded, creating a need for resource integration [1]. There have been several attempts to reconcile this vast and expanding array of services with data and service integration. Many of these approaches have relied on the creation of web-based service portals that seek to integrate and simplify data collection analysis with a wide variety of available tools [2-4], while other efforts have focused on service and data integration through the use of browser-enabled interoperability between services, data providers and even desktop applications [5-7].

The sheer scope and power of data and service integration portals and browser add-ons is also one of the main obstacles to their wide acceptance, since many users rarely need to use more than one or two services (e.g. BLAST and Entrez search) and lack the necessary training in bioinformatics to navigate easily through interconnected repositories of data and services [1]. Still, a wide range of practicing biologists must routinely perform relatively simple manipulation, editing and processing of DNA and protein sequences on a daily basis. To perform these routine manipulations, this substantial segment of users has resorted to proprietary desktop software, like DNAStar or the GCG Wisconsin Package [8,9], ingenious bookmarking of specific web servers, or to services that integrate several tools for sequence manipulation, like the Molecular Toolkit or the Sequence Manipulation Suite (SMS) [10,11].

Web-based sequence editing toolkits like SMS have enjoyed wide acceptance because they provide a simple interface for many routine sequence manipulation tasks and because, running on JavaScript, they are essentially platform independent. Nonetheless, the use of JavaScript results also in some limitations, like the inability to access files on the client computer, which forces the user to rely on copying and pasting data in text format. This does not only add overhead and complicates the organization and storage of data and analysis results, but it also requires that the user have access to raw text data, which may not be the case due to the specific handling of native file formats by the operating system. Last, but not least, the use of JavaScript requires embedding in a HTML file, which many users may find difficult to implement, thus reducing the likelihood of community-based code expansion. To address these shortcomings here we introduce BioWord, an extensive suite of sequence manipulation tools integrated within the familiar Microsoft Word interface. Using a macro-enabled document template, BioWord provides direct and easy access to an array of tools for sequence manipulation, allowing the integration of functionality and data storage within a single interface. Its object-oriented design, implemented in the standard scripting Visual Basic for Applications (VBA) language, facilitates customization, and its integration into a well-known interface provides the means for efficient code-sharing and development.

## Implementation

### Class structure

**Figure 1:**
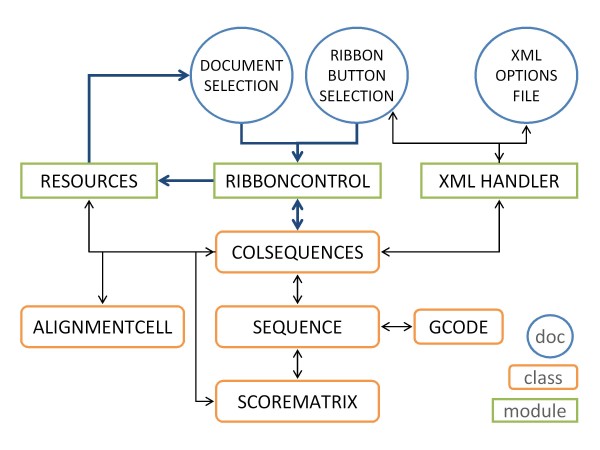
**Schematic representation of the main BioWord control flow (bold blue arrows) and the interplay between classes and modules (black arrows).** Classes are represented by rounded boxes and modules through squared boxes. The design structure is geared to decouple the basic classes (Sequence and ColSequences) from the Microsoft Word interface. A normal workflow starts with the user making a selection on the document and clicking one of the buttons on the BioWord ribbon. The RibbonControl module captures the click, parses the user selection, instantiates a ColSequences object and calls the appropriate ColSequences method. Control returns to the RibbonControl module upon completion of the required processing, and a call is made to the Resources module to implement the output process.

The object-oriented implementation of BioWord is based on two main classes that handle the key elements BioWord is designed to process: sequences and collections of sequences (Figure 1). The Sequence class is used to hold and process DNA, RNA and protein sequences. To simplify the architecture, an instance variable in the class determines sequence type (either DNA/RNA or amino acid sequence) and the sequence itself is stored as a character string. During instantiation, the Sequence object determines its type according to a user-specified percentage of nucleic acid characters [A, C, G, T/U]. The class thus consolidates access to the methods and properties that can be used to process biological sequences and cross-checks their applicability according to the specific sequence type. The ColSequences class is designed to handle the serial manipulation of sequences and those applications requiring the simultaneous processing of more than one sequence, such as sequence alignments. Based on the native VBA *Collection* object, the ColSequences class is used to store multiple Sequence objects and define processing methods for them. The ColSequences class thus implements generic methods to serialize single-sequence processes (e.g. reverse) and methods to process the collection as a whole, such as computing a position-specific frequency matrix (PSFM) or implementing a greedy pattern search on a collection of sequences. Because single sequences are instantiated as unitary ColSequences objects, this class effectively centralizes all interactions with Sequence objects. This primary class outline is complemented by three additional classes that define generic objects used in sequence processing. The GCode class implements a variable genetic code model able to incorporate codon usage data, and is used in any operations involving DNA-protein translation or the use of codon usage tables (e.g. detection of Open Reading Frames (ORF)). The AlignmentCell class is designed exclusively for use in alignment algorithms and provides the means to define all the relevant fields in a dynamic programming alignment matrix. Finally, the ScoreMatrix class consolidates the different scoring rules used by pattern matching and alignment algorithms into a single type of object (the scoring matrix) which defines the methods used to set and use scoring matrices in these different settings.

### Module structure

The class structure is functionally wrapped within a module structure that basically handles the interface with Microsoft Word document objects. This design strategy is aimed at decoupling the basic BioWord objects from their running environment, thus avoiding the need for derivation of specialized classes when, for instance, specific output formats are desired. The RibbonControl module handles basic communication between the ribbon, the ColSequences objects and the document. It contains the methods the ribbon buttons are linked to, thereby defining the functionality of the ribbon. Upon capture of a button-click event, the RibbonControl parses the user selection, instantiates the necessary ColSequences object and calls the appropriate ColSequences method to process the selected sequences, thus implementing the fundamental control flow of BioWord (Figure 1). The RibbonControl module also centralizes reception of ColSequences methods results and calls the appropriate method to handle their output according to sequence type and formatting options. Methods for output generation are stored in the Resources module, which handles both the specific format (e.g. FASTA or table) and destination of the output. BioWord allows output to be redirected to the clipboard, a new document, immediately following the selection or overwriting it. In addition, the Resources module defines a broad set of handy functions to manipulate both sequence and non-sequence objects, like sorting or removing duplicates from a collection. Two additional modules complement this basic module architecture. The XMLHandler module manages the interaction with the XML Options file (which defines the option fields for BioWord) and handles the loading, saving and updating of the option fields available in the ribbon.

### Integration, editing and distribution

BioWord is written fully in VBA and is compliant with the Visual Basic 6 standard, thus maintaining backwards compatibility with earlier versions of Microsoft Office. Due to its explicit detachment of basic Sequence and ColSequences classes, which encode sequence processing functionality, from the document interface, the core of the code is readily adaptable to all versions of Microsoft Word supporting VBA, as well as to other Microsoft Office programs, such as Excel. BioWord is fully encapsulated within a macro-enabled (.dotm) template facilitating its distribution and installation through the use of the Open XML format [12]. The code and the XML Options file are embedded within the .dotm structure, which also contains the ribbon stored as a XML file. BioWord code can be edited with any text editor or, more conveniently, within the integrated VBA editor of Microsoft Word. The XML Options file and the XML ribbon can be edited also with any text/XML editor. For convenience, the XML ribbon can also be edited with the freely available Open XML Custom UI Editor [13].

## Results and discussion

**Figure 2:**
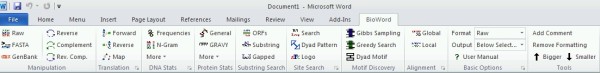
**The BioWord ribbon.** Functionally related tasks are grouped in separate tabs. Additional buttons and tab-specific options can be accessed through the boxed arrow icon located at the bottom right of tabs.

BioWord provides an easily accessible and expandable toolkit for the manipulation and editing of biological sequences embedded within a Microsoft Word ribbon (Figure 2). To facilitate user interaction, the ribbon is divided into several functional groups that are discussed in the following sections.

### Format and sequence manipulation

In its current implementation, BioWord can parse and convert to and from three widespread formats for biological sequences: FASTA [14], GenBank Flat File [15] and bare/raw sequence. Conversion buttons are available in the *Manipulation* group, along with reverse and complement (DNA/RNA) buttons, but output conversion can also be made implicit by setting the *Format* option of the *Basic Options* group to the desired format.

### Translation and sequence statistics

**Figure 3:**
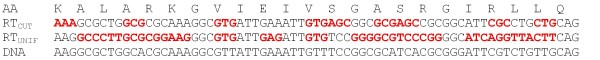
**Comparison between reverse translation of the *Escherichia coli *K-12 MG1655 LexA protein (NP_418467) assuming a uniform codon distribution (*RT***_***UNIF***_**) and using the *E. coli *codon usage table (*RT***_***CUT***_**) supplied by the Codon Usage Database [**[16]**].** Red bold indicates deviation from the real DNA sequence shown at the bottom.

BioWord features frame-dependent DNA to protein translation and translation maps using different genetic codes, as well as reverse translation using a variety of approaches (Figure 3). Reverse translation can be performed assuming a uniform codon distribution and using IUB characters to encode redundancy, or following a codon usage table, provided by the user in GCG Wisconsin Package format, as generated by the Codon Usage Database [8,16,17]. Basic statistics for DNA and protein sequences are also implemented in this distribution of BioWord. Among other, the toolkit can provide n-gram statistics and window-based analyses of DNA %GC content, as well as protein-specific indices, such as the GRAVY score [18]. The output for these analyses is generated in table format and can be readily pasted into spreadsheet software for graph generation.

### Search methods and consensus logos

**Figure 4:**
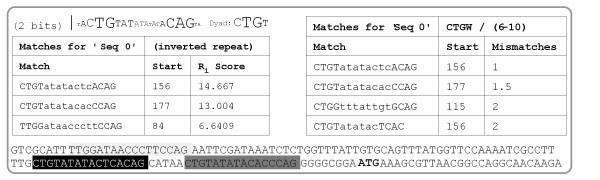
**Sequence search on the *E. coli *K-12 MG1655 *lexA *(b4043) promoter region (125 bp upstream of the translation start point, shown in bold), using several of the search methods implemented in BioWord and a collection of known *E. coli *LexA-binding sites [**[22]**].** (Top left) Consensus logo representation of the LexA-binding site collection and its dyad motif. (Bottom left) Table format results for a *Dyad Pattern* search using with the dyad motif and 6-10 variable spacer. The overall *R*_*i*_ score is the sum of individual dyad scores. (Right) Table format results for a *Gapped* substring search using with CTGW and WCAG as substrings, maximum mismatch of 2 and 6-10 variable spacer. The overall score is the sum of dyad mismatch scores. (Bottom) Superimposed results for a pattern *Search* using the LexA-binding motif. In this output mode, the grey-scale shading intensity that highlights located sites is based on the information score (*R*_*i*_), with darker shades indicating higher-scoring sites.

String and pattern-based search methods comprise a significant part of BioWord’s functionality. The output for search methods can be overlaid on the sequence (highlighted) or provided in table format. BioWord provides a simple-to-use ORF search tool, which can maximize ORF length alone or combined with a supplied codon usage table from a reference genome. Basic string search methods (*Substring Search*) enable mismatch-based search for sequences and the ability to specify variable spacers in *Gapped* search. Mismatch-based search can operate on DNA sequences incorporating IUB redundancy codes or apply standard (e.g. BLOSUM62) scoring matrices to weigh matches in amino acid sequences. Pattern-based methods (*Site Search*) provide a more robust approach to sequence search by incorporating PSFM models and using Shannon’s mutual information or relative entropy derived methods to score putative sites [19-21]. PSFM models are built from collections of sites and/or IUB consensus sequences provided by the user either in raw or FASTA sequence format. Like mismatch-based methods, pattern-based methods allow (*Dyad Pattern*) searching for variable spacer motifs based on direct or inverted repeats of a provided pattern (Figure 4).

BioWord also exploits the ability to handle PSFM models to address a pressing need in the representation of sequence motifs. It is well known that consensus sequences are an unsuitable representation of sequence motifs because they omit information on the importance of consensus bases and the relative frequency of non-consensus bases at each position of the motif [23]. Sequence logos are able to integrate these two missing elements, together with the consensus, in an encapsulated representation and are therefore a superior and preferred method for the representation of sequence motifs [24]. Unfortunately, sequence logos are graphic elements and many authors continue to use consensus sequences to represent motifs in order to avoid the need for additional figures or to allow in-text discussions about the motif. BioWord provides a solution to this problem by allowing the representation of sequence motifs in text format using the consensus sequence, but depicting simultaneously its information content. For instance, the LexA-binding motif of *Escherichia coli*[22] would be represented as . In this representation (the consensus logo), the vertical bar character is used to represent the y-axis scale, with the maximum value, in bits, provided next to it. The height of the consensus letter at each position corresponds to the positional information content of that position (using either mutual information or relative entropy measures). This representation does not provide frequency information of non-consensus bases and, therefore, a sequence logo should be used preferentially whenever possible. Nonetheless, the consensus logo provides the means to convey information about positional conservation in text format and its use of information theory units allows straightforward comparison of motifs (e.g. the LexA-binding motif of *E. coli* can be directly compared to that of the α-Proteobacteria [25]).

### Motif discovery and alignment

**Figure 5:**
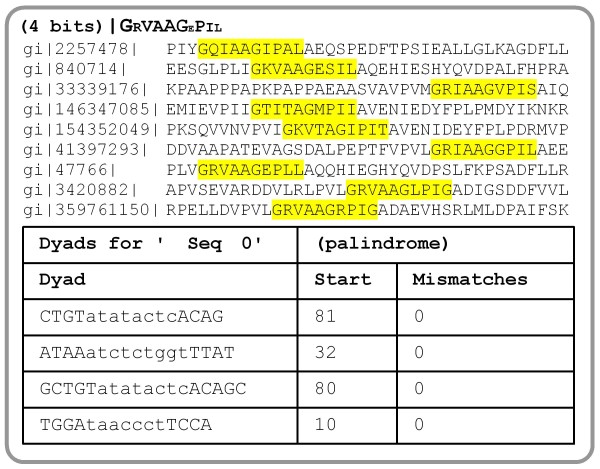
**(Top) Motif discovery with *Gibbs Sampling *on a set of LexA protein sequences from different bacterial phyla.** Instances of the discovered motif are highlighted on the sequences using the superimposed output option. The detected 10 amino acid-long motif shown in the consensus logo is centered on the well characterized Ala-Gly cleavage site of LexA [31]. (Bottom) *Dyad Motif* search on the *E. coli* K-12 MG1655 *lexA* (b4043) promoter region (see Figure 4), with 4±1 bp dyad, 8±1 bp spacer and 2 allowed mismatches. The reported score is the sum of dyad mismatch scores.

**Figure 6:**
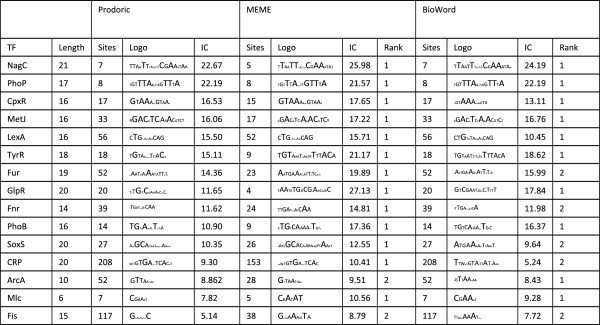
**Benchmark of BioWord and MEME motif discovery against *E. coli *transcription factor binding sites downloaded from the Prodoric database [**[30]**,**[32]**].** Each binding site was expanded 50 bp on each side using adjacent *E. coli* genome sequence to generate motif discovery input data. Motif discovery results for BioWord are from the greedy search algorithm. MEME searches were conducted using the San Diego Supercomputing Center (SDSC) MEME web service. For both MEME and BioWord, parameters were made as similar as possible: Prodoric site length, one site per sequence, search given strand only, 3 reported motifs. In BioWord, the iteration number was set to 100. For both methods, the motif shown corresponds to the best fit with the Prodoric motif. The transcription factor (TF) and length of its binding motif are provided in the leftmost columns. In each block, the number of sites (available in the database or reported by the method), the consensus logo and the information content (IC) of the motif are shown. The rank of the best-fitting motif (based on e-value for MEME, information content for BioWord) among the three reported motifs is also indicated. All logos are in the same scale, with cell height corresponding to 2 bits of information. Input sequences for motif discovery and site sequences for all reported motifs can be found in Additional file 1 .

BioWord supports several methods for motif discovery. The user can apply a greedy search strategy or Gibbs sampling to a collection of unaligned DNA or protein sequences [26,27] in order to locate underlying motifs of a given length (Figure 5). Both greedy search and Gibbs sampling are initialized randomly and iterated as many times as specified by the user. The reported motif is the one yielding larger information content across all iterations. The current distribution of BioWord also incorporates a *Dyad Motif* search tool. This is a string-based motif search tool for bipartite motifs that reports all the occurrences of direct or inverted repeats with a maximum number of mismatches on the dyad and variable spacing (Figure 5). In addition, the package incorporates global and local pair-wise sequence alignment by implementing the Needleman-Wunsch and Smith-Waterman algorithms [28,29]. Memory management and computing power are constrained in BioWord by the use of Microsoft Word-embedded VBA code. As a result, computationally or memory intensive methods in BioWord, such as motif discovery cannot match the capabilities of equivalent specialized resources, like MEME [30]. Nonetheless, benchmarking of the BioWord greedy search algorithm on several known *E. coli* transcription factor-binding motifs indicates that BioWord motif discovery algorithms can provide results that are qualitatively comparable to those obtained by MEME, locating the known motif in nearly all instances (Figure 6), and alignment of relatively long sequences (e.g. 2,500 aa) can be performed seamlessly within BioWord.

## Conclusions

BioWord integrates many commonly used methods for sequence manipulation and editing in a single add-on for Microsoft Word, providing a powerful and easily-accessible toolkit for biological sequence processing in an environment familiar and accessible to most practicing biologists. Among other functions, the current version of BioWord implements bi-directional translation, ORF detection, consensus logos, Gibbs sampling and several powerful sequence search methods. Its simple class structure and modular design based on an accessible object-oriented language (VBA) facilitate customization, code expansion and sharing. Together with its encapsulation in an environment that most students know well, these features make it also a powerful educational instrument.

## Availability and requirements

**Project name:** BioWord

**Project home page:**http://sourceforge.net/projects/bioword/

**Operating system(s):** Microsoft Windows

**Programming language:** Visual Basic for Applications (VBA)

**Other requirements:** Microsoft Office 2007 or higher

**License:** GNU GPL

## Competing interests

The authors declare that they have no competing interests.

## Authors’ contributions

LA implemented the BioWord code and manuals. DMF implemented a legacy Visual Basic version of BioWord for Office 2003/XP and was involved in the design of the basic BioWord class structure. LA and IE designed the class and module structure of BioWord. IE conceived the idea, defined the functionality of BioWord, oversaw code development and drafted the manuscript. All authors read and approved the final manuscript.

## Supplementary Material

Additional file 1 **Motif data for several transcription factors as downloaded from the Prodoric database and motif discovery results for MEME and BioWord (greedy search).**The file contains the native sites from Prodoric and the expanded sites (±50 bp) used as input for motif discovery, as well as the sites reported by MEME and BioWord for the best of three reported motifs. Click here for file
